# Acceptability of an In-home Multimodal Sensor Platform for Parkinson Disease: Nonrandomized Qualitative Study

**DOI:** 10.2196/36370

**Published:** 2022-07-07

**Authors:** Catherine Morgan, Emma L Tonkin, Ian Craddock, Alan L Whone

**Affiliations:** 1 Translational Health Sciences University of Bristol Medical School Bristol United Kingdom; 2 Movement Disorders Group Bristol Brain Centre North Bristol NHS Trust Bristol United Kingdom; 3 School of Computer Science Electrical and Electronic Engineering University of Bristol Bristol United Kingdom

**Keywords:** sensor, Parkinson disease, wearables, cameras, acceptability, smart home, behavior change, multimodal home-based, qualitative

## Abstract

**Background:**

Parkinson disease (PD) symptoms are complex, gradually progressive, and fluctuate hour by hour. Home-based technological sensors are being investigated to measure symptoms and track disease progression. A smart home sensor platform, with cameras and wearable devices, could be a useful tool to use to get a fuller picture of what someone’s symptoms are like. High-resolution video can capture the ground truth of symptoms and activities. There is a paucity of information about the acceptability of such sensors in PD.

**Objective:**

The primary objective of our study was to explore the acceptability of living with a multimodal sensor platform in a naturalistic setting in PD. Two subobjectives are to identify any suggested limitations and to explore the sensors’ impact on participant behaviors.

**Methods:**

A qualitative study was conducted with an inductive approach using semistructured interviews with a cohort of PD and control participants who lived freely for several days in a home-like environment while continuously being sensed.

**Results:**

This study of 24 participants (12 with PD) found that it is broadly acceptable to use multimodal sensors including wrist-worn wearables, cameras, and other ambient sensors passively in free-living in PD. The sensor that was found to be the least acceptable was the wearable device. Suggested limitations on the platform for home deployment included camera-free time and space. Behavior changes were noted by the study participants, which may have related to being passively sensed. Recording high-resolution video in the home setting for limited periods of time was felt to be acceptable to all participants.

**Conclusions:**

The results broaden the knowledge of what types of sensors are acceptable for use in research in PD and what potential limitations on these sensors should be considered in future work. The participants’ reported behavior change in this study should inform future similar research design to take this factor into account. Collaborative research study design, involving people living with PD at every stage, is important to ensure that the technology is acceptable and that the data outcomes produced are ecologically valid and accurate.

**International Registered Report Identifier (IRRID):**

RR2-10.1136/bmjopen-2020-041303

## Introduction

### Background

Parkinson disease (PD) is a slowly progressive neurodegenerative disease that leads to multiple potential movement-related and non–movement-related symptoms, such as muscle rigidity, slowness of movement, tremors, and sleep disturbance [1].

PD symptom progression is currently measured using clinical rating scales [2], which have flaws, including a *snapshot* nature that misses the hour-by-hour fluctuations of PD symptoms and a suboptimal capture of in-home symptoms that differ from those in the clinic [3-5]. Recent work has focused on continuous, longitudinal *passive* (not requiring any active input from the participant) monitoring with technological sensors to produce very frequent objective measurements of specific parameters (eg, gait and tremor). Digital sensors have the potential to measure PD movement-related symptom fluctuations and, potentially, disease progression while a person lives their life freely without external intervention (*free-living*). However, the acceptability (the extent to which the target population considers an intervention to be appropriate based on their cognitive and emotional responses to it [6]) of various types of passive technological sensors for free-living at home has not been widely explored in PD.

### Prior Work

#### Passive Sensors

Ideally, any outcomes being measured by technological devices would be clinically relevant, associated with health-related quality of life [7], and developed in collaboration with the patient population under study [8,9]. We have limited knowledge about the perspectives of people with PD regarding the acceptability of multimodal sensors, including cameras, in the home environment.

Wearable devices (devices equipped with sensors used to measure, process, or analyze health indicators from the person wearing them [10]) have received significant research interest in the study of PD [11-14]. Worldwide, multiple groups have used wearables to detect symptoms of PD, such as bradykinesia and dyskinesia, in a free-living setting [15-17]. They can be worn on different locations on the body, including the wrist, lower back, and lower limbs.

Thus far, the development of wearables to measure PD symptoms has largely focused on the ability of devices to measure symptoms, with relatively few quantitative or qualitative studies exploring the acceptability of the devices [18-21]. Qualitative research methodology, such as the use of interviews or focus groups, can complement other types of intervention development work such as pilot studies [22] by allowing opportunities for a deeper understanding of factors that could impede or facilitate the implementation of an intervention. Acceptability work examining wearables has found that they are acceptable for periods of several days to a few weeks [23], especially if study participants perceive that they will benefit from the provided results [24] or if there is a high caregiver burden associated with their PD [25]. However, one of the groups used questionnaires with free-text responses to explore the experience of 1 week of bilateral wrist-worn wearables at home and found that comfort and wearability decreased over this period [26]. Compliance with wearable device use over periods of several months can be relatively high [23,27]; however, concerns have been raised through qualitative work that social acceptability [18,19] and issues with usability [20] are barriers to wearable sensor use in PD. AlMahadin et al [21] conducted semistructured interviews followed by focus groups with people with PD (who had not been required to have experience in wearable research) to scope the patient perspectives related to the preferences and requirements of wearable device design. They found that the body location felt to be most acceptable for wearable use over longer periods was the wrist and that the participants did not have concerns related to the device visibility or data privacy [21]. The mentioned studies examined various aspects of wearable acceptability in PD; however, this study is unique in that it examines the acceptability (through qualitative work or otherwise) of wearables alongside other sensors at home in PD.

In addition to wearables, other sensors such as cameras can be used to quantify PD symptom parameters. Video data, processed in such a way as to reduce identifiability (eg, Open Pose [28]), have been used to measure symptoms such as resting tremor, finger tapping [29], and sit-to-stand [30]. Many people with PD already have passive smart home–type technologies in their homes [31]. Cameras and other sensor modalities can be used in multiple heterogeneous sensor systems (described for the purposes of this paper as *multimodal* sensor platforms, meaning multiple different types of sensors) providing data from various sources in a range of formats. Multimodal sensing has been shown to be more accurate in distinguishing between PD and control based on common activities of daily living [32,33] than unimodal sensing, which has been used to distinguish different severities of PD [34], predict medication status [35], and detect symptoms such as freezing of gait [36]. Multimodal sensor platforms are also being increasingly used to detect and quantify activities of daily living in-home settings [37]. Given these uses, multimodal sensing presents an opportunity to better understand how the multiple fluctuating symptoms of PD interact with the (at times, complex [38]) daily life in PD compared with unimodal sensing. However, there is an as of yet unmet need for studies exploring how participants with PD feel about living with privacy-preserving cameras or multimodal sensor platforms in their daily lives.

**Figure 1:**
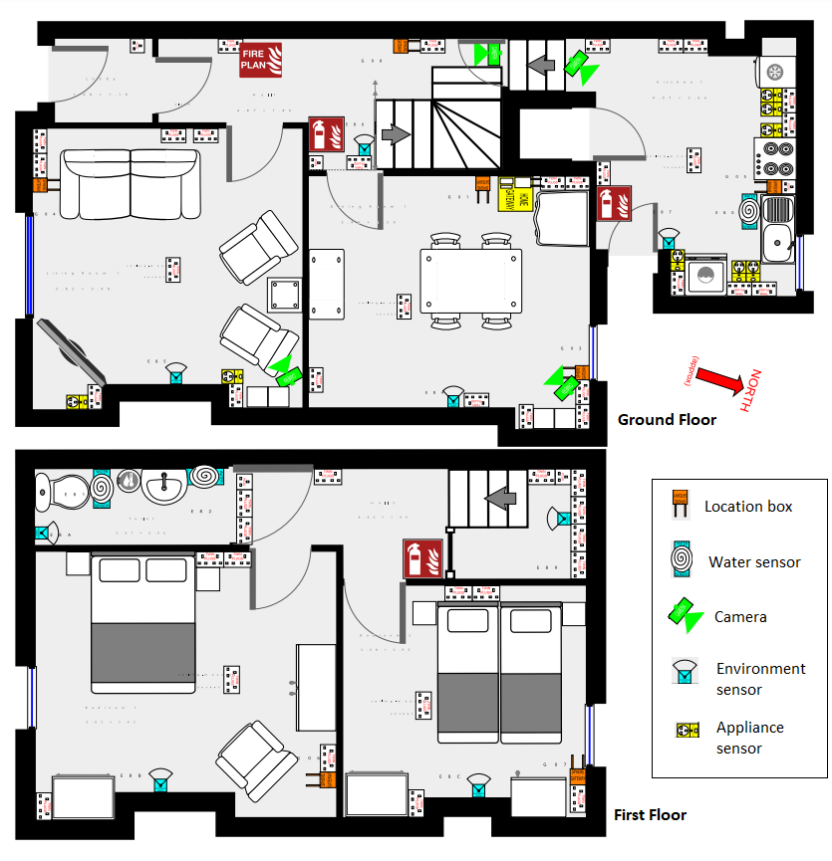
Sensor layout in the study setting.

Our group developed a multimodal sensor platform using (1) *ambient sensors* (embedded in the environment) such as appliance sensors, mains electricity use detection, water pipe use quantification, environmental sensors detecting humidity and temperature, and others (Figure 1 shows the device layout in the study setting, and Table 1 shows the details of sensor capabilities); (2) wearable devices; and (3) cameras producing privacy-preserving video data. By *privacy preserving*, for the purposes of this paper, we imply a privacy-enhancing silhouette-based obfuscation method [39] for preprocessing video data to reduce identifiability. The platform of relatively inexpensive multimodal sensors can be scaled to multiple homes. Its use has been found to be acceptable in the general population [40] and in specific medical conditions [41]; however, no work has been conducted to investigate acceptability in PD. Exploring multimodal sensor acceptability could deepen our understanding of how people with PD interact more widely with technology [42].

Living with new technology may lead to conscious or subconscious adjustments in users’ behaviors or activities [43]; however, currently, there are few studies investigating this in PD, with a limited number of studies focusing on specific aspects, such as the wearables’ impact on daily activities [18].

Understanding the impact of a multimodal sensor platform on the behavior and lives of people with PD can help design such platforms that limit sensor-derived behavior changes to enable accurate measurement of symptoms and activities in one’s own home.

**Table 1:** Sensors used in this study and their sensing capabilities.

Sensor	Sensing capabilities	Details of placement
Wearable (triaxial accelerometer)	Movement Indoor localization	One worn on each wrist
Received Signal Strength Indicator sensors	Movement Indoor localization	On walls usually behind furniture
Privacy-preserving video cameras	Silhouette outline 2D and 3D bounding boxes around participant	On walls of downstairs communal rooms above the eye level
Environmental sensors	Humidity Temperature Light Pressure	On walls above the eye level
Passive infrared sensors	Detecting movement	On walls above the eye level
Appliance sensors	Use of kettle, toaster, television, washing machine, refrigerator, and microwave	Attached to appliance plug and plugged into an electrical socket
Main electricity use sensor	Use of mains electricity	Attached to an electricity meter
Mechanical flow sensors	Use of toilet and taps in bathrooms	Attached to water pipes inside cupboards

#### High-Resolution Video

High-resolution video data can be used to add additional objective evidence about a symptom at the time when another passive sensor is collecting data, evidence that is called *ground truth.* Understanding how someone experiences high-resolution video capture, including how it may alter behavior, could inform design choices in future ground-truthing work for PD. There is some evidence of positive attitudes toward high-resolution video capture at home in PD, where examples of cameras that could be used were discussed [44]; however, minimal further acceptability studies have been conducted with patients with PD who have directly experienced free-living high-resolution video recording.

### Goals of This Study

In this study, a cohort of participants with PD and healthy volunteer controls stayed in a home-like setting equipped with a multimodal sensor platform (described previously). Throughout their stay, they were passively sensed by the sensor platform. The ground truth was obtained at prespecified and limited times using high-resolution video cameras. The participants mostly lived freely during this time. We then explored this experience in depth using interviews and set the comments in the context of the participants’ prestudy attitudes toward technology, as well as their age, disease stage, and other characteristics.

The primary objective of this study was to explore the acceptability of in-home multimodal passive sensors, as well as intermittent high-resolution video data capture, in people living with PD.

Two further subobjectives were as follows: (1) to identify any proposed limitations, controls, or other suggested alterations to the multimodal sensor platform, with a focus on PD; and (2) to specifically identify any self-reported behavior changes resulting from the use of technological sensors in this study.

## Methods

### Setting

A fully furnished 2-bedroom terraced house was embedded with a wide range of passive and unobtrusive sensors (described in Table 1). The wrist-worn wearables used in the sensor platform comprised a triaxial accelerometer with a medium-width strap made from blue- or yellow-colored silicone rubber and a pin buckle clasp, and one device was worn on each wrist. The participants wore a second device on each wrist as part of the research team’s collaboration with IXICO, a UK-based imaging and digital biomarker analysis company. However, for this qualitative work, only the impact of our group’s colored devices was explored. The other sensors were mounted statically (eg, on walls), with no interaction required between the participant and these devices. The cameras were wall mounted above the eye level in each of the 4 *communal rooms* in the house: kitchen, hall, dining room, and living room. This house has been used in many previous studies involving human participants.

Participants were recruited to live freely in this house for a period of 5 days and 4 nights while they were passively sensed. They were able to come and go from the house and continue their activities of daily living. Between 1 and 3 prearranged hours per day, high-resolution video data were recorded from the communal rooms of the house. They were visited by a researcher on only 1 occasion between arrival and departure (apart from the 2 pairs who were visited twice for technical reasons). The study data were collected between October 2020 and June 2021; during this time, several COVID-19–related national lockdowns took place in the United Kingdom, and thus, in some cases, participants were obliged to spend almost all their time in the house.

The participants were given an electronic tablet device that they could use to pause sensor data collection temporarily or permanently or delete data already collected.

Written information was provided to each participant before the study data collection, detailing what the study would involve, which sensors would be used, and what they measured. The participants had at least two telephone or video-conferencing calls with a researcher before participation, during which they had the opportunity to ask questions and discuss their thoughts.

### Participants

**Figure 2:**
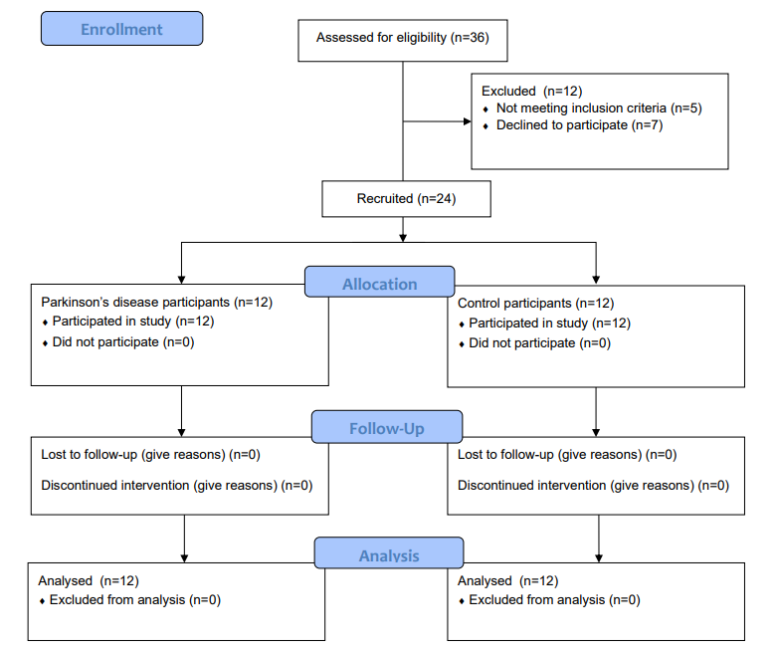
CONSORT (Consolidated Standards of Reporting Trials) flow diagram of study recruitment and attrition.

A total of 24 participants were recruited, 12 (50%) with PD and 12 (50%) healthy volunteer controls (called *control participants* for the purposes of this paper). The CONSORT (Consolidated Standards of Reporting Trials) flow diagram of participant recruitment and attrition is shown in Figure 2.

The participants stayed in pairs in the house, and thus, data were collected from 12 separate 5-day periods. Participants were recruited through convenience sampling, Movement Disorders clinics in the participating National Health Service Trust, our partner charity Cure Parkinson’s, our local Movement Disorders patient and public involvement group, or word of mouth. Written consent was provided by all participants.

### Data Collection

Sensor data were collected continuously (or episodically for high-resolution video data), as described previously.

Semistructured interviews were conducted by a single researcher (CM [MD], a female neurology specialty registrar trained in qualitative interview methodology) in the house on the final day of the study. The participants met and spent time with the researcher on at least two separate occasions before the interview and once in person. They were aware of the research goals of the study and the aim of the interviews. All participants reported that they were comfortable participating in the interviews in their pairs. The interviews lasted between 25 and 56 minutes. The interview topic guide is shown in Textbox 1 and was discussed with the participants before the interviews commenced. Interviews were recorded on a secure encrypted audio-recording device and transcribed verbatim post hoc (transcripts not returned to participants), with field notes made during the interview by the researcher.

**Interview topic guide.**

1. Experience of staying in the study setting
2. Thoughts about the sensors and sensor platform
3. Discussion of the high-resolution video camera data collection
4. Thoughts about the data collected
5. Specific focus on Parkinson disease and the discussion around the sensors in relation to Parkinson disease

A total of 3 subscales of the Media Technology Usage and Attitudes Scale [45] were completed by all participants to better understand how they viewed sensors and technology in their own lives. These subscales included 12 questions related to Positive Attitudes Toward Technology (6 items), Anxiety About Being Without Technology or Dependence on Technology (3 items), and Negative Attitudes Toward Technology (3 items). Each item, listed in Multimedia Appendix 1, comprised a statement scored using a 5-point Likert scale: 5=strongly agree, 4=agree, 3=neither agree nor disagree, 2=disagree, and 1=strongly disagree.

This tool was designed to measure media and technology involvement across a variety of research studies, either as a whole scale or using any subset of the 15 subscales.

### Data Analysis

The audio recordings were listened to multiple times to familiarize ourselves with the data. The transcripts were open coded systematically line by line by CM (using NVivo [QSR International] software [46]) following a flexible and inductive methodological approach. The open codes were reviewed and refined and then grouped into subcategories, which were subsequently grouped into themes without a preexisting coding framework [47,48]. The themes were then reviewed and refined by splitting, combining, and examining the relationships between themes. The final themes were reviewed and agreed upon with a second researcher.

The participants were allocated a study identification for this study according to whether they had PD (*PWP*) or were a control (*C*) participant, alongside a number for their pair, randomly selected from 1 to 12. For example, a person from pair 3 with PD was coded as *PWP3*.

### Philosophical Approach

This study sought to understand participants’ opinions on the sensor platform’s acceptability, recognizing that the results were best understood when set in the participants’ social and experiential contexts. This relativist ontological stance [49] was chosen to reflect the research team’s belief in the subjectivity of reality—that each participant may experience the sensor platform differently and that this experience is best understood by considering contextual factors such as their prior attitudes toward technology and demographics such as age and severity stage of PD (or status as a control volunteer).

Furthermore, to explore the subjective reality experienced by the participants, an interpretivist epistemological approach [50] was used to prioritize the research goal of understanding what the individuals’ views were over an explanation of why they were expressed. The interviews followed a loose topic guide; open-ended questions with no right or wrong answers were used to initiate discussions around the chosen topics (Textbox 1). This approach to the interviews was designed to create an interactive relationship between the researcher and participant, where the multiple potential realities of their experience were explored to produce a detailed, rich, and complex data set. The interview design, combined with the iterative inductive data analysis approach [47] described previously, also aimed to enhance the validity of the data by exploring individuals’ opinions as opposed to solely focusing on what was general and average in the cohort. However, in addition to this individual-level approach [51], some cohort-level quantitative results were produced to illustrate comparative themes and patterns for the reader.

The trustworthiness [52] of this research was carefully considered. Credibility was promoted using interviews of substantial length, where persistent observation from the researcher aimed to deeply prove opinions. Some direct quotes were checked with individual participants to determine the accuracy of their content. In addition, debriefing questions after study participation were designed to triangulate the findings of the interviews using sources collected at different times and in different settings (study setting and participants’ homes) from each participant. This source triangulation of qualitative data, along with the complementary quantitative study results, aimed to reduce individual researcher bias. The collection of demographic and questionnaire data as part of the detailed description of individual participants could enhance the applicability of the study findings to other contexts [53].

### Ethics Approval

Ethics approval was granted by the National Health Service Wales Research Ethics Committee 6 on December 17, 2019, and approval from the Health Research Authority and Health and Care Research Wales was confirmed on January 14, 2020 (reference 19/WA/0051).

Participation in this study was voluntary; participants had the right to withdraw at any stage without the decision affecting their medical care or legal rights. Participants gave informed consent to participate, explicitly agreeing to participate in study interviews that were audio recorded and to the publication of anonymous quotations. Each participant was anonymized by being assigned a unique study identification number, and all directly identifying details were removed from the interview scripts. Data were held securely and processed in line with the General Data Protection Regulation. To avoid issues related to undue influence from one person in the pair toward the other, a separate or joint interview choice was offered.

## Results

### Participant Demographics

The 24 participants were divided into 9 heterosexual spousal pairs (n=18, 75%), 1 pair of close friends (n=2, 8%), and 2 parent-child pairs (n=4, 17%). Each pair comprised 1 participant with mild to moderate PD and 1 healthy volunteer control participant (participant demographics are included in Multimedia Appendix 2). All participants were of White British ethnicity. The cohort with PD had a mean age of 61 years, with the control cohort’s mean age being 59 years. The mean number of years since the PD diagnosis in the cohort with PD was 8.4 years.

Multimedia Appendix 3 demonstrates the participants’ scores on the Media Technology Usage and Attitudes Scale subscale on attitudes; the relevant subscale questions are illustrated in Multimedia Appendix 1.

### Acceptability

#### Privacy-Preserving Cameras

There was a universal sense from the participants that, with informed consent, the collection of privacy-preserving video data from the communal rooms in this setting was acceptable. Indeed, there was a general feeling, articulated by PWP4, that the privacy-preserving video data crossed an acceptability threshold (compared with high-resolution video) for collection in more private rooms:

I wouldn’t mind something monitoring my silhouette in that [the bedroom], but you wouldn’t want color cameras on in the bedroom. I think it’s making sure that it’s not too intrusive.

Sensor acclimatization occurred relatively quickly according to all participants. PWP2 felt “the first day you are more cautious...but within hours you get used to them”; however, for C2 it was “literally five or ten minutes until you get used to it.”

#### Wearables

Overall, the (wrist-worn) wearables were the least acceptable of all the sensors, both in terms of the frequency of issues mentioned by the participants and the noted intrusiveness on free-living. PWP10 mentioned the following:

The only ones [sensors] I was aware of were the ones on my wrist. I, kind of, never even gave the other ones a second thought.

PWP6 went further to say the following:

If there was a system that I didn’t have to do that [wear wearables], that would be better, but, yeah, because the data you’re collecting is important, then, yeah, I would.

In relation to PD symptoms, there were concerns about wearable usability and comfort issues, for example, related to sensitive skin:

I can imagine putting on the wearables when you’ve got the shakes would be a complete nightmare.

PWP7

Put the blue one on too tight, and I woke up with an itchy rash.

PWP8

The participants felt that it would be helpful for them to choose their own strap and device to suit their preferences.

There were mixed feelings about how the wearable devices made the participants feel psychologically. C3 noted the following:

I don’t know how I would’ve felt outside [wearing the wearables]. I think, personally, I would’ve covered them over.”

It was noted that improved aesthetics would be likely to encourage compliance. However, others did not find wearable aesthetics an issue:

I went out in a short-sleeved dress at one point and it just didn’t bother me.

C1

Interestingly, PWP5 felt that the wrist-worn wearables empowered him and that he “felt like James Bond*.*” He said that if someone asked him about the wearables, he would “put some story to it”; he actively enjoyed the sense of feeling different from others by wearing devices for research.

How people outside the study viewed the wearables and their impact on the participants were discussed. PWP8 said that in a regular group she attended, she “had to pull my sleeve up and show them what I’d got on my wrist*.*” However, the idea of worrying about what others thought of the research wearables had not occurred to PWP3:

I’ve got a lot more other things that I’d be more worried about showing someone rather than the wearables.

Multiple participants commented on their experience of wearing the wearables overnight. There were anxieties from 8% (2/24) of participants about the fit of the wearables affecting the data’s usefulness. PWP2 was concerned that one of her wearables had “slipped around and was facing down, practically all night I guess,” and thus, the data may be inaccurate. For some, the wearables themselves disturbed sleep “when you’re turning, because you’re trying to find a way of being comfortable, then the wearables are more noticeable*.*” However, for some, the wearables at night were more tolerable than they had anticipated:

I thought they would really annoy me...but it didn’t.

PWP7

#### Other Ambient Sensors (Environmental, Appliance, Mains Electricity, Water Pipe, and Passive Infrared)

These sensors were universally found to be acceptable in the context of the study, and all participants anticipated that they could live with these sensors for several months in their own homes. C7 articulated this by saying “I mean, we looked at them when we came in, and thought, ‘Oh, that’s where they all are,’ and carried on*.*”

#### Whole Sensor Platform

Approximately 96% (23/24) of the participants found the sensor platform acceptable to live with and would be happy for it to be deployed to their own homes for long periods of up to a year.

The one participant who was uncomfortable with the idea of sensor deployment to his own home, PWP11, had quiescent but long-standing obsessive-compulsive disorder before entering the study, which was reactivated around the time of participation. He felt that completing the diaries and being sensed by technological devices reminded him that he had PD. He said the following:

I think what is not a problem for a few days or for a week would be a different kettle of fish if you were looking at weeks or months on end. And, also, well, it would be a strange feeling to know that your decline into illness is being charted on an ongoing basis. Sometimes it’s good not to dwell on it, not to be too—not to get obsessed about your own health.

However, he also emphasized the importance of appropriate informed consent before deployment:

If you make it clear what’s on the menu, then people can opt in on that.

He also acknowledged the conflict between data impact and research benefit and his discomfort related to the sensors:

Well, it’s balancing its usefulness against its intrusion, isn’t it?

#### Privacy

Approximately 96% (23/24) of the participants stated that they had no significant privacy concerns about the passive sensor data, and the overwhelming response was expressed by PWP10, who said the following:

It comes down to if it’s helpful and useful, then it’s in my own best interests, isn’t it, you know, to say, “Do with it as you wish.”

The reasoning behind this broad acceptance of privacy risks was varied. PWP3 felt that “Me getting out of bed and getting dressed as a silhouette wouldn’t be interesting to anybody anyway.” PWP11 noted that personal data was collected in other circumstances with more limited consent than in this study:

In terms of personal information, you can walk down any street in city A and you’ve got CCTV recording your movements without asking your permission and digging a lot less anonymous data out.

PWP12 made the point that he perceived that the sensor data of the nature collected in this study would be difficult to use in a negative way against the participants, in contrast to, for example, genetic profiling and the impact on insurance premiums:

I think it’s completely different than DNA or blood types, or...I think, now, if you could connect those two together, then that might be more of a problem.

However, one of the participants (C3) expressed concerns about video data getting into the wrong hands and being disseminated via the internet:

The only problem is that, and it’s me being neurotic, I suppose, the fact that you hear all these things on the internet, people putting videos, and things, on the internet, and I know they’re [directly-identifiable study data] not on the internet, and I know they’re secure, and all this, but that’s always been in the back of my mind.

#### Intermittent High-Resolution Video Data Capture

When directly questioned, all participants found the high-resolution video data acceptable during this study. Before participation, some participants were nervous about it:

We heard about the cameras, and we weren’t quite sure how intrusive they would be. So, we probably were more guarded than we are now, you know, we’re more at ease now [having finished the study].

PWP6

However, PWP12 noted that they found the high-resolution video recording “less intrusive than I thought it was going to be, which is good, yeah*.*”

### Optimizing Sensor Platform Design

#### Limitations

All participants were happy with the privacy-preserving cameras in the communal rooms—kitchen, dining room, living room, and hall.

Regarding other camera locations, of the 24 participants, 16 (67%; n=8, 50% with PD, and n=8, 50% controls) were happy to consider having privacy-preserving video data captured in the bedroom. Approximately 31% (5/16) made the caveat that they would ideally have some control over such collected data (eg, to be able to switch the data collection off). The positives of collecting sensor data in bedrooms were noted by several participants (eg, capturing important bedroom-specific symptoms and activities):

There is a lot, lot to capture for a person with Parkinson’s that happens in the bedroom. So, that’s where the person gets dressed, that’s where the person has their nightmares.

C5

For those not wanting cameras in bedrooms, reasons given were related to privacy around dressing, personal hygiene, and sexual activity, where a camera was felt to be “too invasive...I’d feel too worried about that” (C9).

PWP9 mentioned that he felt the bedroom cameras would not capture interesting data from him:

I agree with the thing about that first 45 minutes or hour, or whatever, can be quite difficult, but I think, for us, we tend to get up and go downstairs into the kitchen, and get our breakfast.

He felt that whether cameras were deployed in bedrooms or bathrooms should be managed on a case-by-case basis, depending on how people lived and used the rooms in their houses.

Interestingly, 21% (5/24) of the participants would consider cameras deployed in bathrooms in their own homes. This was a controversial topic, with the remaining 79% (19/24) of participants feeling that it was not acceptable.

I think that personal hygiene is your personal hygiene, and I think it should be done behind closed doors.

C5

There were acknowledgments of the conflict between data usefulness and privacy; for example, PWP12 felt the following:

If it’s limiting the research not doing it [collecting data from bedroom or bathroom], or it’s helpful to do it, then I think as long as you have the ability to control it

then it may be acceptable

Some participants discussed ways of mitigating the privacy invasion of cameras. For instance, C5 said they would be happy for researchers “to do it [record sensor data from bedroom or bathroom] on specific nights, so that you [participants] had a break away from it.”* *Interestingly, this was countered by C9, who felt that she would be happier with continuous data collection if she could gain a fuller understanding of the data; she wanted first to “have a look to see what that [sensor data] looks like so I could now how invasive it feels, I think it wouldn’t make a difference whether there was a holiday [sensor-free period] or not.”

Some participants were categorical in that at least some sensor-free space in the house was needed for in-home deployment:

I think if there was a case of the cameras were in every room of the house, people would be very uncomfortable.

PWP3

You then wouldn’t get people acting normally.

C3

#### Sensor Controls

Of the 24 participants, 10 (42%; n=6, 60% with PD) felt that *participant*-operated sensor data collection controls were appropriate for the following reasons: participant-led control would be better than predefined *camera-free times* so that the episodes of worse symptoms would not be missed, to collect data of specific activities that participants perceived to be important for researchers to capture (“you could switch it on and off as and when you felt it’s something valuable”), and to turn the sensors off at important times to the individual:

If I was being completely honest, I think taking my clothes off in front of a camera that was on would possibly make me feel quite vulnerable, I wouldn’t be comfortable with it, but once I’d then got my pyjamas on, then it wouldn’t bother me again

C5

The nature of such a control device was discussed by 8% (2/24) of participants, and both felt that having a handheld device would be ideal for periods of poor mobility:

Some sort of control which was on your person, because in our bed, this morning, particularly, I was, to get out of bed is a real struggle sometimes.

PWP3

PWP3 and C3 noted that the touch screen interface of the electronic study diary did not suit them: C3 found touch screens difficult to use, in part because she is left-handed, and they both felt that “touch screens and Parkinson’s don’t really make sense” because of the impact of having tremors and reduced fine motor dexterity*.* They suggested that the sensor control device should have a large “button, really, because people can’t manage with the remote controls [with small buttons], can they?”

Notably, none of the participants paused or deleted any sensor data during this study.

Interestingly, there were some strong opinions held by the 8% (2/24) of participants who preferred sensor controls to be held by the researchers. PWP5 felt this because of the following:

I think if you’re [researchers] in control of it, I think that’s better, yeah, because it’s going to get done, isn’t it? If I’m in control of it, I might not film it.

PWP12 made the additional comment that he would not want to unwittingly introduce bias in the data captured:

You could become in the habit of always controlling it on, I don’t know, when you’re cooking, or whatever, just as an example, so it becomes a really skewed view of what you’re doing, if you’re not careful.

PWP9 made the point that being able to control the sensor data collection may increase awareness of the sensors and reduce the unobtrusiveness of the platform:

Wouldn’t want to control—I’d want them just to be there, because then I think I’d just forget that they were there, and that—I wouldn’t want to have it on my mind the whole time.

In particular, the idea of having a control mechanism to delete data after it had been captured was not seen as important by C3, who said that the ability to “delete information, and stuff, which, to me, is silly, because if you’ve recorded it, you keep it in*.*”

#### Practicalities

At least 2 participants raised concerns about their occupations, which would not allow wrist-worn devices while at work (health care professional and construction industry).

The look and sound of the nonwearable sensors were discussed on several occasions. To acclimatize to the sensors and reduce the reminders that they were being sensed, there was a desire for the devices to blend into the background of the room. PWP6 mentioned the following:

There’s no light that tells you it’s on, and I think that’s an important thing...if they suddenly went—lit up, you’d think, “Oh, the camera’s on,” and you’d change, but you tend to forget about it.

However, C8’s view of the light was that it felt normal:

You’ve got all sorts of alarms in houses these days with little red lights in the corner, and sensors, you know, the odd little red light up there doesn’t seem—it’s just normal these days.

The possibility that sensors might emit sound was also identified as a possible barrier to acceptability by PWP10, who found the lack of noise from the sensors to be positive:

It’s all silently in the background, you’re not aware of it. It’s not as if there’s whirring and clanging machines, or anything, is it?

The location of the cameras in the corners of rooms above the eye level was mentioned as positive for acceptability and as having a low impact on free-living by PWP8, who said the following:

It’s not evident, is it? You haven’t got a great big camera staring you in the face.

#### Focus on PD Outcomes

Some participants had recommendations for specific symptoms or times in the day that should be prioritized for data collection in future research. These included sensing both day and night as “The night-times are the times where you can get a truer picture of what’s going on...” (C4), and over multiple days to weeks as opposed to intermittently:

It varies how good you are...You don’t want a good day, in a way, you want a bad day so you can see how people manage.

PWP5

Other outcomes that participants felt should be a focus of future research were the ability to climb stairs, outdoor activity, and the impact of menstruation on PD.

#### Behavior Change

Although all participants asserted that they had not consciously changed their actions for the passive sensors, it became evident from the unsolicited interview responses that behavior changes had occurred.

The participants felt a sense of responsibility toward capturing “good” data, which, at times, translated into behavior change. For example, PWP7 mentioned the following:

Did take my watch [wearable] off this morning, I think it was, then realised I’d left something in the bedroom, so I picked it up and carried it with me...because I had three on and one off.

They worried the data would not be complete, and C10 mentioned they were more “conscious of how long we were out [of the house]” so as not to reduce the amount of data captured. Generally, there was also a heightened awareness of the activities that would be helpful for the research team. For example, C3 said the following:

My concern was we weren’t doing enough for you, actually physically moving around.

On a more human level, the perception of being sensed had subtle impacts on the participants’ interpersonal and private behaviors. C3 felt that they and PWP3 felt less free to be tactile with each other in the study:

We’re more touchy/feely normally, and cuddly, and things, which we didn’t do.

PWP5 said that they “made sure I didn’t go around with no clothes on.”

However, none of the 24 participants felt that they had to escape from or *trick* the sensors during the study, and they largely felt able to live as they would normally. For example, C4 stated that they were “really just transposing our life from our own house into, more or less, what we’re doing in this situation...we were, more or less, oblivious to being recorded, I think.”

Participation in this study led participants to wonder what the researchers would think of their behavior from the sensor data. The reactions ranged from the severity of their PD symptoms, with PWP10 “trying to open something and it wouldn’t come open. I think I was thinking ‘This’ll look bad. I can’t even open a paper bag,’” to a more general sense that researchers may query or misinterpret their activities of daily living. For example, PWP4 said the following:

There was one evening when [C2] was giving my back a massage upstairs, and I said, “God, what’s this going to look like on the sensors?”

However, the use of intermittent, preagreed times of high-resolution video data capture introduced a marked difference in the awareness and behavior of some participants compared with the continuously used *background* sensing. C11 articulated the following:

You’re more hyperaware if you’re being observed [by cameras capturing high-resolution data]. It almost makes you on your best behavior.

They were backed up by others, including C12 who felt they “certainly wouldn’t have a disagreement if you thought the cameras were on you, I don’t think*.*” Several participants mentioned that they would try to capture data that they perceived to be helpful to the research team during these 2-hour epochs. PWP4 said that “if anything, you want to put a show on, rather than just sitting inert,” and C5 felt that they made “sure that it was PWP5 [their study partner] that was doing the activities so that you were capturing their movements.” The prearranged times of high-resolution data capture were intrusive enough that participants appeared wary that the cameras were filming at other times:

There’s been a couple of times where PWP5’s gone right up to the cameras and, “They are, they’re filming, they are filming, C5.” And I’m like, “No, they’re not, PWP5, don’t worry about it.”

C5

However, conversely, PWP8 denied any impact on their behavior:

I just knew they were there and just forgot about them. even forgot about the times when I was supposedly being videoed.

## Discussion

### Principal Findings

This work has found that it is acceptable to use multimodal sensors, including wrist-worn wearables, cameras, and other ambient sensors passively, in free-living patients with PD but that the least acceptable sensor was the wearable device. There were several suggested controls and limitations related to future sensor deployment in people’s own homes, especially for camera use. Behavior changes during this study were noted by the study participants, which may have been related to being passively sensed. Recording high-resolution videos in a home setting for limited periods was considered acceptable.

### Acceptability

#### Multimodal Sensor Acceptability, Including Privacy-Preserving Cameras

There were very few concerns related to data confidentiality or security, largely as the participants judged that the benefits of advancing research into PD outweighed any personal concerns about data misuse, which is in line with other research looking at the motivations behind providing personal data for research [54]. The person who expressed concern about the privacy of camera sensors, C3, felt that she had always been more aware than other people she knew about the possibility of covert video recording, for example, when she visited hotels. Beyond this, she did feel that she trusted the security of the data management by our research group. Interestingly, C3 gave the highest score on the *negative attitudes* subscale of the Media Technology Attitudes and Usage Questionnaire; therefore, she placed herself as feeling very negative toward technology. Her *anxiety/dependence* subscale score was very low, indicating that she felt not at all dependent on technology in her day-to-day life. These attributes may have contributed to the articulation of stronger views about privacy than the other participants.

This is the first qualitative study to compare wearable acceptability with that of other sensors. Compared with the other devices, wearables were the least acceptable sensor type in this platform, both in terms of the frequency of issues identified and the intrusion on free-living activities. This was related to practical issues around comfort, aesthetics, usability with PD symptoms, and the impact of the devices on sleep. This contrasts with some of the literature on the experience of using wearables in PD, where other groups have reported good acceptability of wrist-worn devices [21,55], albeit with reports that wearable acceptability decreased over time [26]. An important finding from this study was the variation in the wearables’ psychological impact, with reports varying from positive descriptions of a sense of empowerment or a willingness to show them off to negative feelings, including a desire to conceal them from other people. The fear of wearables socially identifying someone’s age, disease, or disability has been found by other groups [18,20,27] and should be a factor in device design, for example, concealing research-grade sensors in a wrist-watch (eg, off-the-shelf devices such as the Apple Watch [56]). It is possible that wearables were the least well tolerated as they are the only sensors that require direct participant interaction—increasing the intrusion on free-living compared with ambient sensors—and that further work toward a more seamless transition between digital sensing and physical wearable use [57] could help improve wearable acceptability in PD. The inclusion of health-tracking features in wearables has been found to improve their acceptability related to passive sensing [58,59]. This is possibly related to moving away from a reliance upon, or conversely, a suspicion of, technology and toward a relationship of trust through confidence in technology as a helpful instrument with which to visualize symptoms and therefore better understand our bodies [60].

One of the participants (PWP11) had a strongly negative response toward the entire sensor platform, which reminded him of his identity as a patient. He did not feel it would be tolerable to live with for longer than a few days, partly because of the impact on his mental health. This contrasts with the generally positive impressions of other participants. PWP11 had conflicting results on the Media Technology Attitudes and Usage questionnaire, with a low average score on the *positive attitudes* subscale (indicating that he was not overly positive about technology) but a high average *anxiety/dependence* and low average *negative attitudes* subscale scores (suggesting that he is more dependent on and not very negative toward technology); therefore, this questionnaire was not helpful in interpreting his experience. Psychiatric comorbidity alters how someone interacts with technology, and conversely, trends in the use of technology can predict whether someone has specific psychiatric disorders [61]. When designing a platform for people with PD, it is important to take special care to consider how its sensors may influence psychiatric symptoms such as depression, apathy, anxiety, and cognitive dysfunction (as well as how data may be affected by these factors) as these are all possible symptoms of PD. The potential utility of in-home sensing needs to be balanced against the risks to individual participants.

This is the first study to examine how people with PD experience video data collection while living freely in a home-like environment. Interestingly, the *privacy-preserving* cameras, which we imagined the participants may have felt negatively about, were well-accepted by our participants, and a high level of trust in the research team may have facilitated camera acclimatization. The reported time taken to acclimatize to cameras varied between minutes and hours, which is an important consideration as the time when behavior may be altered may need to be removed from the final data analysis of future studies. The person who reported acclimatizing quicker (C2) had a more positive attitude toward technology than the person who took longer (PWP2), adding weight to the importance of evaluating an individual’s technological attitudes when designing further similar studies.

Obstacles to camera deployment to homes included instances where fellow home-dwellers cannot give informed consent for this data collection (eg, children or people with cognitive impairment). In such cases, it would be important to unpick both the acceptability and appropriateness of the use of any sensor, particularly those devices whose data outputs are not fully anonymous.

The ambient sensors (environmental, water pipe, appliance, mains electricity use, and passive infrared sensors) were well tolerated and posed no acceptability issues to our participants, indicating that their deployment to people’s own homes would be reasonable.

#### Acceptability of Intermittent High-Resolution Video Data Recording

Intermittent high-resolution video recording was found to be acceptable to all participants while they were free-living in this setting, although some participants had been wary of how they would feel before the study.

Given the need to ecologically validate sensor data findings in the real world [62], we anticipate more camera sensors will start to be used for this purpose; this study lends support that this is an acceptable study design choice if full informed consent is gained from participants.

#### Design Adaptations Suggested for Home Sensor Deployment

To increase the acceptability of home-based use of these sensors, several controls and limitations were suggested by our participants, especially for camera use. This included the following: camera-free time, especially related to recording in the bedroom; no cameras in bathrooms (according to 19/24, 79% of participants); and some camera-free space in the house.

Two-thirds of the participants were happy to consider cameras in their bedrooms for research purposes, largely driven by the motivation to understand symptoms that occur in bedrooms, which are currently poorly quantified. This finding is supported by other studies exploring the acceptability of home-based video recording [44]. When considering camera placement within these more *private* (bed or bath) rooms, it is important to recognize the potential risks to human dignity, especially related to vulnerability and sense of self [63], and efforts should be made to minimize the amount and identifiability of the data collected in these rooms. It should be noted that the methods used to produce silhouette video data are ways of working to protect the privacy of participants through camera use. Other methods have been described by Li et al [39], including the use of a body avatar or a point-light representation.

The question of whether sensor data collection control was needed (or not), as well as who should operate it (participant or researcher), drew mixed responses from our study participants. A bespoke system whereby participants were offered the option of who, if anyone, would control sensor data collection, including how to pause or delete data, may be a future route for free-living passive data-sensing studies.

The participants were interested in how the sensor platform design could be optimized to minimize intrusion in day-to-day life. Perhaps unsurprisingly, they generally wanted devices that would blend into the background of their homes without sounds or lights to highlight that they were there. They were concerned about how their friends and family would interact with and feel about the sensors in the homes they shared with study participants. This is echoed in similar findings from another group that highlighted the importance of social acceptability and aesthetics of technological sensors in PD [64]. A key point was that some participants simply would not be able to wear wrist-worn wearables during the day because of their occupations. To ensure the generalizability and inclusion of the younger working population of people with PD, these kinds of limitations need to be considered in future sensor platform design.

### Participant Behavior Changes

#### Related to Passive Sensors

The semistructured interviews drew out several different behavior change examples from participants regarding passive sensors. There was feedback that the participants were aware of how they may be viewed, or even judged, by the research team for their symptoms or what they did in the house. This awareness of how others perceived themselves is important to recognize when trying to capture naturalistic behavior: a heightened awareness may have caused some of the conscious or subconscious behavior modifications described in the results and may also affect how the participants view themselves [65]. The aim of our group and many others was to record natural free-living behavior; however, if our passive sensors alter subconscious (or conscious) behaviors, this needs to be carefully considered in future study design and data interpretation.

#### Related to High-Resolution Video Recording

Our results showed how the use of intermittent video changed some of our participants’ behaviors in a seemingly marked way at time, so the use of video ground truthing in real world settings should be judicious and targeted to symptoms which are accurately evaluated by clinicians watching the videos. Perhaps it should also be agreed that participants will either not be informed when high-resolution video is being recorded, or recording could be done over longer periods for participants to acclimatize to the sensors and normalize their behavior as much as possible.

### Needs and Opinions Related to PD

Unmet needs related to the quantification of particular PD symptoms and daily activities identified by this study’s participants included a 24-hour view of symptom fluctuations; nighttime symptoms, including nocturia and sleep; the impact of menstruation on symptoms; activities outside the house; and mobility on ascending and descending stairs. The everyday management of PD symptoms and daily activities is complex and fine-tuned, and others have called for a technology-assisted outcome measure design to address this complexity [38]. We echo this and advocate that people with PD be involved in the design of any system measuring free-living home-based technology platforms to measure aspects of their disease.

### Study Limitations

This study included a small group of people with mild to moderate PD who had a relatively wide age range (46-74 years), all of whom were of White British ethnicity. The sample size, lack of candidates with severe PD, and absence of ethnic diversity indicate that we cannot generalize the study results to the wider population of people living with PD. In addition, a selection bias is likely to be present; those who offered to participate in a study such as this may be more positively disposed toward technology, and thus, we cannot assume that the largely positive opinions on technology acceptability reflect the views of all people living with PD. The study population’s education level and prior experience with digital or assistive technology should be collected in future studies. Living alongside someone with PD is likely to influence the control participants’ opinions related to sensors, and thus, it is important to bear this context in mind and not to assume that *control* opinions are entirely independent of PD; rather, they are more reflective of the next of kin and carers of people with PD. The study location in a home-like setting was different from one’s own home. One could speculate that being in such a location heightens awareness of being sensed, thus affecting behavior and activities more than being in one’s own home. Furthermore, the relatively short duration of 5 days may not have been enough time for the sensors to feel *normal* to the participants; thus, the amount of behavior change may diminish in studies over longer periods.

### Conclusions

This study showed that it is broadly acceptable to live with multimodal sensors, including wrist-worn wearables and cameras, for 5 days in a free-living environment and that most study participants would be happy to consider these sensors’ deployment in their own homes. However, the least well-tolerated of the sensors were the wearables, and the participants suggested several limitations on passive sensor use at home, including sensor (especially camera)–free time and space. A significant subset of the study participants wanted to see the ability to control home-based sensor data collection, for example, in the form of being able to pause the sensors. Participants reported a range of behavior and activity changes during the study, some of which may have been related to the passive sensors used. When considering the validation of sensor data in a home environment, the use of high-resolution video cameras to provide a ground truth was found to be acceptable in this study. A future direction of qualitative work could be to evaluate how people living with PD feel about the sensor platform in their own homes.

These findings, in the context of other research in this field, should help inform design choices for studies involving passive sensing in-home environments. People living with PD should play an active role in developing such sensor platforms and studies, especially when choosing symptoms that should be measured.

## Appendix

Multimedia Appendix 1 The Media Technology Usage and Attitudes Scale subscale questions on attitudes and anxiety about dependence on technology.

Multimedia Appendix 2 Participant demographics and details about Parkinson disease.

Multimedia Appendix 3 Results of the Media Technology Usage and Attitudes Scale subscale scores for each participant.

## Abbreviations

CONSORT: Consolidated Standards of Reporting Trials
PD: Parkinson disease
