# Supplementary material for: Acceptability of an In-home Multimodal Sensor Platform for Parkinson Disease: Nonrandomized Qualitative Study
Source: JMIR Hum Factors. 2022 Jul 7;9(3):e36370. doi: 10.2196/36370 (PMC9305404; doi:10.2196/36370)
Supplement: Multimedia Appendix 1 [file humanfactors_v9i3e36370_app1.docx]

Media Technology Usage and Attitudes Scale sub-scale questions on attitudes and anxiety about/dependence on technology.

| **Sub-Scale** | **Questions** |
| --- | --- |
|  |  |
| **Positive attitudes** | I feel it is important to be able to find any information whenever I want online. |
|  | I feel it is important to be able to access the internet any time I want. |
|  | I think it is important to keep up with the latest trends in technology. |
|  | Technology will provide solutions to many of our problems. |
|  | With technology anything is possible. |
|  | I feel that I get more accomplished because of technology. |
| **Anxiety/dependence** | I get anxious when I don’t have my cell phone. |
|  | I get anxious when I don’t have the internet available to me. |
|  | I am dependent on my technology. |
| **Negative attitudes** | New technology makes people waste too much time. |
|  | New technology makes life more complicated. |
|  | New technology makes people more isolated. |
