# Supplementary material for: Acceptability of an In-home Multimodal Sensor Platform for Parkinson Disease: Nonrandomized Qualitative Study
Source: JMIR Hum Factors. 2022 Jul 7;9(3):e36370. doi: 10.2196/36370 (PMC9305404; doi:10.2196/36370)
Supplement: Multimedia Appendix 2 [file humanfactors_v9i3e36370_app2.docx]

Participant demographics and details about Parkinson disease.

| **Participant** | **Age range** | **Gender** | **Years since PD diagnosis** | **Hoehn and Yahr Score (off medications)** |
| --- | --- | --- | --- | --- |
|  |  |  |  |  |
| PWP1 | 50-54 | Female | 7 | 3 |
| PWP2 | 65-69 | Female | 19 | 3 |
| PWP3 | 55-59 | Male | 11 | 3 |
| PWP4 | 60-64 | Female | 17 | 2.5 |
| PWP5 | 55-59 | Male | 18 | 3 |
| PWP6 | 70-74 | Male | 1 | 1.5 |
| PWP7 | 45-49 | Female | 0.5 | 1 |
| PWP8 | 70-74 | Female | 5 | 3 |
| PWP9 | 55-59 | Male | 11 | 2 |
| PWP10 | 60-64 | Male | 3 | 1 |
| PWP11 | 70-74 | Male | 2 | 2.5 |
| PWP12 | 55-59 | Male | 6 | 2 |
| C1 | 60-64 | Female |  |  |
| C2 | 20-24 | Male |  |  |
| C3 | 55-59 | Female |  |  |
| C4 | 65-69 | Male |  |  |
| C5 | 50-54 | Female |  |  |
| C6 | 70-74 | Female |  |  |
| C7 | 70-74 | Female |  |  |
| C8 | 75-79 | Male |  |  |
| C9 | 55-59 | Female |  |  |
| C10 | 55-59 | Female |  |  |
| C11 | 60-64 | Female |  |  |
| C12 | 55-59 | Female |  |  |
