# Supplementary material for: Acceptability of an In-home Multimodal Sensor Platform for Parkinson Disease: Nonrandomized Qualitative Study
Source: JMIR Hum Factors. 2022 Jul 7;9(3):e36370. doi: 10.2196/36370 (PMC9305404; doi:10.2196/36370)
Supplement: Multimedia Appendix 3 [file humanfactors_v9i3e36370_app3.docx]

Results of Media Technology Usage and Attitudes Scale sub-scale scores for each participant.

|  | **Technology Attitudes Sub-Scales** | | |
| --- | --- | --- | --- |
| **Participant** | **Positive** | **Anxiety and Dependence** | **Negative** |
|  |  |  |  |
| PWP1 | 27 | 7 | 12 |
| PWP2 | 19 | 11 | 12 |
| PWP3 | 20 | 3 | 12 |
| PWP4 | 16 | 3 | 12 |
| PWP5 | 23 | 11 | 12 |
| PWP6 | 26 | 6 | 7 |
| PWP7 | 26 | 11 | 9 |
| PWP8 | 21 | 7 | 12 |
| PWP9 | 27 | 5 | 15 |
| PWP10 | 22 | 6 | 10 |
| PWP11 | 19 | 10 | 8 |
| PWP12 | 23 | 14 | 10 |
| C1 | 25 | 10 | 12 |
| C2 | 22 | 10 | 10 |
| C3 | 23 | 3 | 15 |
| C4 | 21 | 6 | 11 |
| C5 | 21 | 10 | 8 |
| C6 | 21 | 4 | 12 |
| C7 | 26 | 7 | 8 |
| C8 | 24 | 9 | 9 |
| C9 | 21 | 10 | 12 |
| C10 | 22 | 8 | 10 |
| C11 | 20 | 4 | 10 |
| C12 | 20 | 11 | 12 |
